# Managers’ perspectives on their role in implementing fall prevention interventions: a qualitative interview study in Norwegian homecare services

**DOI:** 10.3389/frhs.2024.1456028

**Published:** 2024-09-27

**Authors:** Siv Linnerud, Maria Bjerk, Nina Rydland Olsen, Kristin Taraldsen, Therese Brovold, Linda Aimée Hartford Kvæl

**Affiliations:** ^1^ Department of Rehabilitation Science and Health Technology, Faculty of Health Sciences, OsloMet - Oslo Metropolitan University, Oslo, Norway; ^2^ Division for Health Services, Norwegian Institute of Public Health Oslo, Norway; ^3^ Department of Health and Functioning, Faculty of Health and Social Sciences, Western Norway University of Applied Sciences, Bergen, Norway; ^4^ Department of Ageing Research and Housing Studies, Norwegian Social Research (NOVA), OsloMet - Oslo Metropolitan University, Oslo, Norway

**Keywords:** qualitative research, implementation, fall prevention, homecare services, health services research, leadership, management

## Abstract

**Introduction:**

The implementation of fall prevention interventions in homecare services is crucial for reducing falls among older adults and effective leadership could determine success. Norwegian homecare services provide home nursing, rehabilitation, and practical assistance, to residents living in private homes or assisted living facilities. This study aims to explore how managers in Norwegian homecare services experience implementation of fall prevention interventions and how they perceive their roles.

**Methods:**

We conducted 14 semi-structured individual interviews with managers from different levels of homecare services in five city districts. The interviews were transcribed verbatim and reflexive thematic analysis was used to analyze the material.

**Results:**

The analysis resulted in three main themes: (1) understanding organizational mechanisms to facilitate new practices, (2) practicing positive leadership behavior to facilitate implementation, and (3) demonstrating persistence to sustain implementation. Our results showed the importance of clear leadership across all levels of the organization and the value of devoting time and utilizing existing systems. Managers described using recognition and positive attitudes to motivate employees in the implementation process. They emphasized listening to and involving employees, providing trust, and being flexible. However, the implementation process could be challenging, highlighting the need for managers to be persistent.

**Conclusion:**

Managers at all levels play an important role in the implementation of fall prevention, but there is a need to define and align their specific roles in the process. Understanding how to use existing systems and influence through positive leadership behavior seem to be vital for success. Recognizing the demanding nature of implementation, managers emphasized the importance of systems for long term support. The study findings may influence how managers in clinical practice engage in the implementation process and inform future researchers about managers’ roles in implementation in homecare services.

## 1 Introduction

Falls are recognized as a global health hazard. Every year, one-third of adults aged 65 and above experience falls (1). These falls can lead to reduced health, fractures (2), and severe injuries (3), making falls the second leading cause of unintentional injury-related deaths (4). The economic implications of falls represent 1% of healthcare costs in high-income countries (1). We have solid evidence on effective methods to prevent falls among older adults, including interventions such as strength and balance training (5, 6), medication review including modification and/or deprescribing (7, 8), and adjustments to home hazards (9, 10). In 2022, the World Falls Guidelines were published as a global initiative to prevent falls (1). These guidelines recommend actions for identifying falls, categorizing the level of fall risk, and providing specific recommendations for different levels of fall risk (1).

The adoption of research evidence and guideline recommendations on fall prevention is particularly important in primary care and homecare services, where there are high incidences of falls (1, 11). Norwegian homecare services constitute the lowest level of care in the Norwegian healthcare system, providing services such as nursing, rehabilitation and practical assistance to inhabitants residing in private homes. As the government strives to promote aging in place (12), homecare services face increased demands to deliver diverse health and care services to vulnerable inhabitants (13). This necessitates a commitment to implement and providing evidence-based services, which also requires healthcare professionals to adhere to new practices. Employees within these services hold a variety of professional training, from individuals without health related training to assistant nurses, registered nurses, physiotherapists and nurse practitioners (14), indicating various levels of competencies. Homecare services further contend with challenges in recruitment, high levels of sickness absenteeism, and turnover (14).

The implementation of research evidence and clinical guidelines is widely recognized as a complex process influenced by barriers and facilitators operating at different levels (15). Known barriers to the successful implementation of guidelines include a lack of awareness of research evidence, negative attitudes, and limited time available for competence enhancement (16). In a systematic review examining the implementation of fall prevention in residential care facilities, common barriers included staff feeling frustrated and helpless, limited skills and knowledge, and poor communication (17). The findings also underscored leadership-related barriers such as leaders lacking quality improvement skills and failing to listen, provide support, or supervise the implementation process (17). Lack of support from managers, together with resistance leadership, i.e., behaviors and attitudes from leaders or managers that oppose or hinder the implementation of new initiatives or changes, has been shown to have a negative impact on the implementation of research evidence (18).

Leadership is considered one of the most important contextual determinants for success in implementation science (19–21). Strong leadership behavior, such as encouraging, motivating, inspiring, and challenging the team to produce their best work, has been suggested as an important facilitator influencing the implementation of research evidence (22). In a recent meta-synthesis, Clavijo-Chamorro et al. (20) highlighted the importance of managers expressing their engagement in the implementation process. Managers who used reminders, encouragement, and training motivated employees (20). A systematic review synthesizing evidence on the leadership behavior of managers associated with employees’ application of research evidence (21), identified change- and task-oriented leadership behaviors as important for successful implementation. These behaviors seemed to influence the implementation climate and how the organization operated. Managers play an essential role in shaping organizational commitment and readiness for change. They influence employees’ willingness and ability to adapt by fostering a shared sense of readiness through consistent messaging and actions (23–26). A systematic review showed that middle managers in primary care influence the implementation climate and therefore play a crucial part in facilitating the adoption of evidence-based practices (27). Middle managers are employees who oversee frontline staff and report to senior management (28). According to the middle-managers theory, middle-managers perform four key roles in implementation processes: (1) disseminating information through passing on necessary information to employees, (2) integrating and synthesizing information about the implementation, (3) bridging the gap between strategy and daily operations through identifying necessary tasks and providing employees with required tools, and (4) advocating for research evidence and engaging employees to implementation (28).

Only a few studies have explored how managers experience their role when it comes to preventing falls (29–31). A knowledge translation project from Australia found that clinical nursing leaders recognized the risks and anxieties involved in taking on an additional leadership role (31). Simultaneously, managers acknowledged the numerous pressures on the system and highlighted the importance of innovation at the local level (31). In an evaluation study on telecare, leadership support and involvement was found to be success factors for screening for fall risk among community-dwelling older adults (29). Furthermore, in a study on implementation culture, managers roles and styles were found to positively influence factors during the implementation phase, in a study from a Norwegian community (30). Previous research has been primarily conducted in the contexts of primary care and hospital environments. To our knowledge, no such studies have been conducted in homecare services.

Despite the importance of effective leadership in the process of implementing research evidence and guidelines (20–22), we lack sufficient insight into the role of managers in the implementation of fall prevention in primary care and homecare services. Accordingly, there is a need for more research to understand the specific contributions and challenges experienced by managers when implementing fall prevention interventions. In this study, we aimed to explore how managers in Norwegian homecare services experiences implementation of fall prevention interventions and how they perceive their roles.

## 2 Material and methods

### 2.1 Study design

In this experiential-oriented qualitative interview study, we explored the experiences and perspectives of participants through individual semi-structured interviews and reflexive thematic analysis based on Braun and Clarke (32). Throughout this study, the view of knowledge is contextualized, shaped by the environments and surroundings in which the knowledge arises and evolves, as outlined by Creswell (33).

### 2.2 Context of the study

The context for this study was the homecare services in five city districts of Oslo municipality, the capital of Norway. The municipality of Oslo holds 634,000 inhabitants and is divided into 15 city districts. Each city district functions as its own administrative unit and is free to organize its services according to local needs to provide health and care services to its residents. The services provided include nursing, rehabilitation, practical assistance, and activity centers for residents of private homes or care facilities. Typically, Norwegian homecare services are structured into teams or departments based on geographical areas (14), overseen by managers responsible for large workforces (34). The number of management levels varies across districts, and responsibility differs according to manger level, with low-level managers overseeing daily activities, middle-level managers implementing top-level plans, and top-level managers setting strategic goals and policies (35). The five districts in this study had three to four management levels, including multiple low-level managers. For instance, in one district, departments were organized geographically, with four levels of management: team nurses overseeing daily tasks, local managers reporting to higher-level managers, and all ultimately reporting to the district's homecare services manager. Employees had diverse competencies as reflected by the various levels of training, ranging from individuals without health-related training to assistant nurses, registered nurses, physiotherapists, and nurse practitioners (14). Additionally, the municipality of Oslo has an overarching agency of health, which holds an advisory and coordinating function towards the healthcare services in city districts. This agency contributes to competence enhancement, research and quality development. The study is a part of the FALLPREVENT project (Implementation of evidence-based, fall prevention programs in the health care services: Quality, competency and effectiveness), which aim to implement evidence-based fall prevention programs in municipal healthcare services across Norway (36).

### 2.3 Recruitment

We approached five city districts in the municipality of Oslo that had prior experience in implementing fall prevention interventions and collaboration with the FALLPREVENT project (36). We conducted a purposeful and strategic sampling process, where we selected managers from different management levels aiming for a diverse range of perspectives and experiences. Managers were approached directly by email and encouraged to forward the invitation to potential participants.

### 2.4 Participants

In total, 14 managers (13 female, age range from 32 to 68 years) from five city districts and the municipality's agency for health participated in the study, representing low-level (*n* = 7), middle-level (*n* = 5) and high-level (*n* = 2) managers. Their formal leadership training and managerial experience varied, from having extensive experience to being relatively new to the role. Furthermore, the participants had diverse experience with implementation, both in their current roles and from previous experience as healthcare professionals. Seven of the managers were nurses, six were physiotherapists, and one had a background as a teacher. The number of participants per management level is presented in Table 1 for each city district.

**Table 1:** Number of participants per management level.

Management level	City district 1	City district 2	City district 3	City district 4	City district 5	Agency for health
Low-level (*n* = 7)	1	3	2	1		
Middle-level (*n* = 5)	2	1		1	1	
High-level (*n* = 2)	1					1

### 2.5 Data collection

We conducted all interviews digitally and recorded them using Zoom. The sample size was informed by information power, as described by Malterud (37), which is based on factors such as the study aim, participant specificity, interview quality, theoretical framework, and analytical approach to determine the necessary number of participants. The last eight interviews in particular provided rich and relevant descriptions of the topic explored. The interviews all lasted approximately one hour, and the first author (SL) conducted all interviews. Data were collected between May 2022 and October 2023. After co-creating and testing an implementation strategy for fall prevention (38, 39), the role of leadership and managers appeared as a crucial aspect of the implementation, which we wanted to explore further. In addition to six interviews with managers from the two previous FALLPREVENT studies, two from the co-creation (38) and four from the feasibility study (39), we interviewed an additional eight managers. Interview guides were designed by the authors, and topics explored inn all interviews covered the participants experience with fall prevention, their role as managers in implementation, and their ideal of optimal implementation process to create lasting changes. The interview guides from both the co-creation study and the feasibility study covered a broader range of topics beyond the role of managers in implementation but included relevant topics for the current study. All interview guides are provided in Supplementary File 1.

### 2.6 Data management and analysis

All interviews were transcribed verbatim by the first author (SL). We analyzed the transcripts using the six steps of reflexive thematic analysis outlined by Braun and Clarke (32). To familiarize ourselves with the material, two authors (SL and LAHK) individually read the entire dataset, while two other authors (MB and NRO) read five interviews each. The four authors independently explored patterns before they gathered to discuss analytic ideas and insights gained from the interviews. Subsequently, two authors (SL and LAHK) independently coded the material, by labelling relevant quotes and organizing them into groups. The authors used the HyperResearch 4.5.3 software to manage data and facilitate the coding process. The coding was conducted mostly semantically, using code labels closely related to the meaning of the quotes. The coding process is exemplified in Table 2.

**Table 2:** Example of how codes were coded and grouped during the analysis.

Quote	Code label	Code group	Associated initial theme
*“So, I think it's natural that I am presenting it [the new routine] from the start and explain how to do it, assist if there's anyone who doesn't understand how to do it, ensure that everyone does it correctly, talk about it”*	Managers follow-up of implementation	Understanding of own role in implementation	Active facilitation for implementation in practice
*“I believe she [my manager] trusts me to do what I am supposed to. I don't know if she has any routines for checking if I am doing what I should. […] I don't get the impression that she checks my work, like I kind of have to.”*	Trust-based leadership	Confidence in the leader	Beneficial leadership behaviour and role modelling

After the independent coding, the two authors (SL and LAHK) consolidated their coding process in HyperResearch, ultimately agreeing on ten initial themes. The initial themes were reviewed and discussed with the other two authors (MB and NRO), drawing inspiration from theory of implementation leadership (40, 41), leadership behavior (21) and the middle manager role theory (28). This process resulted in agreeing on nine initial themes. Next, the initial themes were cross-checked with the data, then further refined and expanded in collaboration with all authors during the writing of the manuscript. Following the development and review of the initial themes, the analysis resulted in three main themes and four subthemes. The progression of initial themes into sub-themes and main themes is presented in Table 3, located in the results section. These themes encapsulated the managers experiences and perceptions of their role in the implementation of fall prevention interventions. The main themes and subthemes were further discussed between all authors and subsequently renamed and adjusted during the writing process. As described by Braun & Clark, this recursive process involved revisiting the coded extracts and the entire dataset to refine and validate the identified themes and subthemes (32).

**Table 3:** Presentation of initial themes, sub-themes and main themes.

Initial themes	Sub-themes	Main themes
Demanding implementation at all levels of management	• The role of clear leadership across organisational levels • The potential of devoting time and using existing systems	Understanding organizational mechanisms to facilitate new practices
Active facilitation for implementation in practice		
Utilize existing work processes for implementation		
Leadership in practice: authoritarian to democratic	• The motivating factors of recognition and positive attitudes • The value of fostering trust and support	Practicing positive leadership behaviour to facilitate implementation
Beneficial leadership behaviour and role modelling		
Implementation: a never-ending story		
Competence enhancement as implementation strategy		Demonstrating persistence to sustain implementation
Systematic evaluation and monitoring of process		
Presence of leaders that maintains systematic follow-up		

The four authors (SL, LAHK, MB and NRO) who conducted the analyses possessed varied backgrounds, one was a nurse and the remaining three were all physiotherapists. All authors had extensive clinical and/or research experience on older adults in primary care. Additionally, one of the authors contributed with managerial experience from the academic field. Prior to the study, the first author wrote a research memo to clarify preconceptions that encompassed anticipation of challenging leadership conditions and limited awareness of the implementation process among managers.

### 2.7 Ethical considerations

This study was conducted in accordance with the Helsinki Declaration (42) and received approval from Sikt – Norwegian Agency for Shared Services in Education and Research (Reference number: 362420). Study participation was based on written, voluntary consent. Before each interview, participants were informed about the study aim and the fact that participation was voluntary, and that they could withdraw at any time without consequences. We collected and stored data in accordance with Oslo Metropolitan University's procedures, ensuring data security with appropriate access control.

## 3 Results

In this study including 14 managers, we identified three main themes: (1) understanding organizational mechanisms to facilitate new practices, (2) practicing positive leadership behavior to facilitate implementation, and (3) demonstrating persistence to sustain implementation. Initial themes, sub-themes, and main themes are presented in Table 3.

### 3.1 Understanding organizational mechanisms to facilitate new practices

The first theme underscores the importance of clear leadership across organizational levels and the potential benefits of dedicating time and utilizing existing systems. Managers expressed how their position within the organization directed their role in the implementation process. For effective implementation, managers utilized existing workflows for knowledge and information exchange in practice.

#### 3.1.1 The role of clear leadership across organizational levels

The managers at different management levels expressed varied perceptions of their roles during implementation of fall prevention interventions, but all of them underlined the importance of their active participation in the process. At the same time, several managers described their roles in the implementation of fall prevention as unclear. Interestingly, low-level managers had a clearer perception of their own roles, as they actively engaged in the practical day-to-day aspects of the implementation. They delegated fall prevention tasks to employees in the services, followed up on performance evaluations, and provided education and competency training in fall prevention to employees. They also kept fall prevention on the agenda, as described by one of the managers:

«My role is to be actively present and visible during implementation. It’s essential to demonstrate the importance by physically being there. Sending an email announcing the implementation is one thing but showing my familiarity with the system and emphasizing the importance of daily discussions, especially regarding fall prevention, is another» (Participant 3, low-level manager)

The role of middle-level managers was described as a supportive function for employees during implementation, assisting upon need. They reminded low-level managers about the process, although, articulating their specific involvement could be challenging. One middle-level manager said: *I must help my employees stay focused, so I try to talk about it [fall prevention] (Participant 14, middle-level manager),* illustrating how vaguely they described their own role. High-level managers indicated that middle-level managers were responsible for executing decisions made at leadership meetings, transferring strategy into day-to-day activity. However, high-level managers described this transfer as lacking. When they began involving low-level managers in leadership meetings, decisions were seemingly translated into action more frequently. This suggests that middle-level managers did not always fulfil expectations regarding their role of transferring fall prevention strategy from top level management into day-to-day activity. One high-level manager said:

«Middle-level managers are integral members of the leadership group, carrying significant responsibility. Once decisions are made, their tasks are to proceed with implementation. In theory, this structure works well. However, in practice, I often find that middle-level managers are hesitant to make decisions independently at their level, without reassurance that they have support for those decisions» (Participant 13, high-level manager)

Some low-level managers described how their superiors were not actively driving implementation of fall prevention forward in the areas they expected them to. They lacked insight into local fall prevention guidelines and utilization in practice. Decisions were not consistently followed up, and feedback was lacking, diluting implementation focus. Lack of interest and engagement was perceived as an indication that the implementation was not regarded sufficiently important, as noted by one participant:

«My previous experience is that decisions are typically made during meetings with high-and middle-level managers and then announced as new initiatives. Subsequently, there is often no further inquiry about these initiatives until a year has elapsed. By that time, interest has waned, and the initiative has lost its momentum» (Participant 4, low-level manager)

High-level managers described their role as focusing more on the strategic aspects of implementation activities within the organization's plans and priorities, while also being responsive to political directives. Overall, the lack of clarity and understanding of own roles, particularly the role of middle-level managers within the implementation process, may inadvertently hinder implementation efforts.

#### 3.1.2 The potential of devoting time and using existing systems

Managers emphasized the importance of creating capacity within their department, devoting time, and using existing systems to achieve successful implementation of fall prevention interventions. This involved allocating time for various fall prevention activities and utilizing existing workflows, such as morning meetings and electronic patient journals. Some managers described how regular meetings was used to remind employees about fall prevention, while others used existing arenas, such as dedicated lunch meetings for competence enhancement, to improve understanding and education on fall prevention. One manager said:

«So, what we experience as good arenas for competence enhancement are these brief sessions of about 40 min held in the social zone during the afternoon shift transition. That’s when everyone gathers and when we have a bit of extra time» (Participant 13, high-level manager)

Using electronic patient journals to organize fall preventive tasks for each service user was illustrated by some of the managers as an important success factor. Low-level managers described how they ensured efficiency by allocating specific time for fall prevention tasks, making these activities clear and visible on their work lists in the electronic patient journal. As part of this routine, they emphasized the importance of systematizing how employees identify, register, and assess falls within patient documentation as one low-level manager said:

«We had to create tasks [in the electronic patient journal] for all the service users, with the correct interval, to make them visible [for employees]. Not just talk about having to do this or that, but to (actually) make it visible» (Participant 7, low-level manager).

The high pace of new requirements, practice routines, and the constant need for improvement posed challenges and was perceived as a barrier for the implementation. Day-to-day issues like managing employee sickness and sudden illnesses among service users led managers to prioritize care tasks. Being able to address both such urgent issues, and managing implementation tasks proved challenging, often resulting in planned implementation activities taking a back seat. One participant said:

«The focus [on fall prevention] tends to fade because there are so many other things happening at the same time. When it comes to implementations, it always come alongside other tasks» (Participant 14, middle-level manager).

By utilizing existing platforms and systems, managers maintained their focus on fall prevention and created space for implementation activities, despite interruptions from daily issues.

### 3.2 Practicing positive leadership behavior to facilitate implementation

The second theme embraces how managers may leverage motivating factors such as recognition and positive attitudes, alongside the value of fostering trust and support. Praise and presence were identified as mechanisms managers used for motivating employees to embrace changes. Managers reported using supportive leadership styles to encourage employee adherence to fall prevention interventions.

#### 3.2.1 The motivating factors of recognition and positive attitudes

The managers described how they used positive recognition by giving compliments and praise to increase their employees’ adherence to the fall prevention interventions. Some managers expressed frustration about having few other methods or tools to enhance adherence among employees. One manager described a lack of consequences for non-compliance with routines and the absence of a system to reward compliant behaviors. Alternatively, managers described how they hoped providing attention to correct behavior and adherence, would set a positive example for others. For example, praise was given to employees who used correct procedures for registering fall risks and conducted correct fall prevention interventions. Managers experienced that such compliments empower staff and serve as motivation for improved performance. One of the low-level managers said:

«I believe that praise serves as a motivation. When you receive positive feedback for the work you have done.. I have seen it myself; you are motivated to do an even better job when someone else (positively) recognizes what you do» (participant 7, low-level manager)

Managers stressed the need to show commitment to the implementation process and for themselves to have knowledge about fall prevention. This commitment involved a positive attitude, being present, and having sufficient knowledge to assist when needed. While managers were expected to exhibit expertise in fall prevention interventions and strategies, transparency about their competence was crucial for fostering trust, even in cases of limited knowledge. Managers who collaboratively seek solutions with employees when confronted with uncertainties further facilitated engagement among employees. One of the managers said:

«Being approachable, addressing their queries, and honestly admitting when I don’t have an answer, working collaboratively to find solutions, is how you get them on board» (Participant 3, low-level manager)

The managers reported that their employees in homecare services possessed varying competence around falls and fall prevention activities. Thus, they found it especially important that the managers had good insight and knowledge about falls before starting the implementation. Some managers expressed that their positive attitudes were important in motivating employees for implementation. One of the managers said:

«The other managers and I did not take a negative stance regarding the implementation. Before introducing it to the department, we underwent a training process and observed its operation. By the time we started the implementation process, it wasn't new to me. This enabled me to speak extensively about the benefits, which is my primary focus. The more positive I am, the easier it becomes for other employees to adopt a positive attitude» (Participant 1, low-level manager)

Some of the managers underlined how implementation of fall prevention interventions should be seen as a collaborative effort, where the contribution of every team member was necessary.

#### 3.2.2 The value of fostering trust and support

The managers illustrated and described different leadership behaviors through explicit statements and details about how they performed their roles as managers. In the material, we found examples of visionary leadership behavior, where managers related the implementation of fall prevention to a broader context and emphasized its societal importance. We also found examples of absent leadership, seemingly displaying a lack of insight into day-to-day practices and a lack of knowledge about the implementation process. Furthermore, we identified a few examples of authoritarian behavior, with managers defending their authority, despite expressing a dislike for authoritarian behavior. Nevertheless, most managers expressed a preference for inclusive, supportive, and democratic leadership styles. Democratic leadership behavior was described as attentive listening and actively involving employees, enabling them to participate in and influence the implementation process. One manager said:

«I anticipated significant resistance from the employees [when staring to implement changes]. […] However, I have only received positive feedback. I don’t know what I do.. I ask… I ask them [employees] for their opinion before deciding on how it’s going to be. This way, they can participate in the process and understand both the pros and cons» (Participant 12, low-level manager)

How the managers spoke about the employees also reflected this democratic leadership style. Some managers praised the level of knowledge and competence the employees had and were proud of how they performed their work. Meanwhile other managers talked negatively about employees, describing low level of competencies among employees and they underestimated their employees’ ability to change practice. One manager said: *You must understand what we are facing: we have employees who don't even read emails or have trouble logging on to the computer (Participant 5, middle-level manager*). However, the democratic leadership style reflected encouragement of input and engagement from employees. One manager emphasized the value of not only asking questions but also encouraging employees to pose questions back. The manager said:

«As a manager, you must utilize the knowledge held by your employees, listen to their perspectives, and consider their opinions on what is right and wrong. Engage in open discussions and encourage questions. […] You provide support when you listen to them, hear them out. You also support them when you disagree with them» (Participant 8, middle-level manager)

The managers also described how trust was an important aspect of leadership. Most of them aspired to be managers who trusted their employees and received trust from their own managers. Trust was described as an integral part of the implementation process, as managers were not able to facilitate all aspects of it themselves. Managers trusted employees to follow fall prevention guidelines, document actions, and follow up with patients, as personally overseeing these tasks would detract from their other responsibilities. Being trusted and providing responsibilities, such as fall risk assessment, patient education, monitoring, and documentation, to employees was also a way to foster growth and development. One manager described how trust provided empowerment and security among employees:

«About gaining trust and passing on trust. […] You can’t be a control freak in this role. If a task isn't solved the way I would have done it, it most likely gets solved in an excellent way, just different from what I would have done myself. And that’s important for succeeding with implementation, providing trust at all levels» (Participant 2, low-level manager)

Receiving trust from their own managers whether it is operational, judgmental, or strategic, provided the space to practice the type of leadership managers preferred and the freedom to adapt their management approach, e.g., communication style, adjusting roles and responsibilities, training methods, and feedback, during the implementation process in practice.

### 3.3 Demonstrating persistence to sustain implementation

The third theme highlights how managers’ persistent and resolute efforts were crucial for maintaining the implementation process over time. The managers highlighted the challenges in ensuring sustainability of the implementation and developing a long-term plan for implementation. The implementation process was seen as continuous, requiring resilience and systematic work from managers. Consistently cultivating and reinforcing a shared understanding among all employees about the importance and methods of fall prevention was crucial, ensuring that everyone comprehended the “why” and “how” of fall prevention. Managers experienced that acquiring knowledge about why fall prevention is important and how to do so was the first step toward fostering a change in employees’ behavior, and this understanding should be pervasive across all levels of the services. One of the managers said:

«Having knowledge about what to do is crucial. […] Understanding falls, their causes, and that they're not a natural part of aging is essential information that everyone should have. It helps us comprehend why fall prevention is necessary» (Participant 4, low-level manager)

The managers described how long-term success required them to be patient and have stamina. Managers found it important to have resilience and systematically follow up on the implementation efforts over time. As described by one of the high-level managers:

«They don't give up. They take one step forward and then two steps back, and then they're at it again, all the time… very persistent» (Participant 10, high-level manager)

In the first phase of the implementation process, managers described that their role was to remind employees about falls and how to correctly adhere to fall prevention interventions, as well as facilitating repetition. Sustaining this effort over time proved challenging and exhausting for some. Few districts had established systems for long-term support, such as statistics to monitor fall rates or adherence to recommendations, forcing managers to devise their own methods. The lack of effective systems hindered persistence. While some managers did not see the need for such systems, others stressed their importance for tracking fall data and monitoring the impact of prevention measures. For example, one manager used fall statistics from service users to guide discussions in team meetings or inform decisions on prevention actions:

«Using the fall statistics immediately and observing that it’s in the evening when the person activates the safety alarm or when the [sensors] is triggered. It happens in the evening, not in the morning. That’s when the fall statistics become relevant. In the morning, you've been resting all night, so maybe then you can walk without a walker. But in the evening, that’s when falls occur, so perhaps you should use a simple wheelchair in the evening and move around that way. Then we can observe an immediate effect on your falls.. That’s how I've been thinking we should utilize the statistics..» (Participant 11, middle-level manager).

Through the description, she illustrated how to use statistics and data to monitor implementation and make it relevant for employees.

## 4 Discussion

This study explores explore how managers in Norwegian homecare services experience implementation of fall prevention interventions and how they perceive their roles. Our results revealed how important it was for managers to understand organizational mechanisms to facilitate new practices. Managers described how clear leadership across organizational levels, devoting time and using existing systems were all important in the implementation of fall prevention. Furthermore, we found that managers practiced positive leadership behavior to facilitate implementation, where recognition and positive attitudes motivated for change. Managers emphasized the importance of providing trust and support by listening and involving employees in the implementation process. However, some managers found the implementation process challenging, requiring them to demonstrate persistence to sustain implementation in practice.

Our results indicate how crucial it is that managers at all levels understand their role in the implementation process. The middle-level managers had a particularly important role, being the ones responsible for executing decisions made at leadership meetings, transferring strategy into day-to-day activity. Bridging this gap has been suggested to be one of four roles middle-level managers fulfil according to the theory of middle-level managers (43). However, our results indicate that some middle-level managers did not fulfil this bridging role and lacked actions to drive progress in implementation. Involvement of managers at all three management levels in the implementation of fall prevention was an expectation from the healthcare providers and other managers, which was highlighted as important by the managers to ensure enthusiasm and supportive communication further down in the organization. As emphasized by Aarons et al., ensuring alignment across different leadership levels in healthcare when implementing new practices is crucial for success (24). A systematic review exploring the role of middle-level managers in the implementation of evidence-based practice supports the notion that middle-level managers play a crucial role in facilitating implementation, as their actions shape the implementation climate (27). The important role of managers was also emphasized by the managers in our study.

The integration of implementation activities into existing workflows was emphasized by managers across all levels, as a tool to streamline the implementation process. According to the updated Consolidated Framework for Implementation Research (CFIR) (44), compatibility between the innovation, in this case fall prevention interventions, and the organization's existing workflows is positively associated with implementation success. This could suggest that managers who know how to use existing workflows within their organization could enhance the effectiveness of implementation. Using existing workflows could also prevent extra time spent on implementation (16), provided that the current workflows support healthcare providers in preventing falls.

To facilitate implementation, managers described using positive leadership behaviors, such as providing praise, modelling desired behaviors, and foster trust and support. Allocation of rewards and use of role modelling are suggested mechanisms to embed and reinforce the implementation climate (24, 41). Employing performance appraisal have also been found likely to promote implementation (45). Managers in our study preferred to employ positive leadership behavior, focusing mainly on encouraging and inspiring their employees rather than using negative tools, in the implementation process. Having positive attitudes towards implementation and being able to inspire and motivate have been associated with predicting implementation success (41). However, positive leadership may not be conducive to effective fall prevention implementation due to its emphasis on top-down decision-making, lack of employee involvement, limited innovation, and potential resistance to change (41). Collaborative and participative leadership approaches may be more effective in engaging staff, promoting innovation, and fostering a culture of safety and prevention within the organization. This type of leadership has been related to transformational leadership, where facilitation, support, mentoring and participatory decision making reflects managers’ behaviors in implementation studies (19).

For some managers in our study, maintaining focus on the implementation process over time was challenging due to the lack of systems for monitoring the process. Aarons et al (24). state that what managers measure and control on a regular basis, and what they choose to ignore, influences the prioritization of healthcare providers. Few managers in our study described currently having access to systems to monitor and support the maintenance of implementation of fall prevention intervention or implementation in general. Monitoring practice and providing feedback is an implementation strategy that is likely effective at improving practice (46). Norwegian regulations on Leadership and Quality Improvement in Health and Care Services encourage services to conduct self-evaluation of implementation activities to increase the quality of services (47). However, it is up to each municipality, most often each manager alone, to establish systems that can facilitate such processes. A systematic review summarized organizational leadership as one of the most frequently reported factors that can effectively facilitate the sustainability of evidence-based interventions in public health (48). However, managers in our study had various backgrounds, and a large share of them did not have formal training in leadership and management. Lack of leadership and management competency among managers can influence how they perceive and execute their roles (49). This can again lead to experience-driven decision-making during the implementation process, even when evidence supports alternative best practice (50).

Our findings underscore the critical role of managers at all levels in the implementation process. Managers are in position to influence the success of implementation by maintaining a positive attitude towards the process, staying attentive to its progress, and taking responsibility for its sustainability. It is imperative to comprehend how managers’ specific contributions influence the implementation process and establish clear role allocation within the organization. Our findings could offer valuable insight into how managers in clinical practice should engage in implementation processes. Overall, the results suggest that municipalities should aim to establish monitoring systems for falls prevention and provide managers with incentives for building monitoring capacities within their organizations. Managers should undergo comprehensive training to enhance their competencies in influencing all phases of the implementation process. Finally, future research should rigorously evaluate specific managerial actions to determine their impact on the success of implementation processes.

### 4.1 Strengths and limitations

One of the strengths of this study was the utilization of multiple investigators with diverse backgrounds, bringing different perspectives that enhance the trustworthiness of the results (51). To enhance the validity and transparency of the results, we provided detailed information and thick descriptions of our preconceptions, researchers’ backgrounds, and the research process (52). We recruited managers from different levels of homecare services aiming to ensure a broad variety of perspectives represented, with varying previous experiences and attitudes towards implementing fall prevention. Still, this study was conducted in five city districts of Oslo municipality, where fall prevention had been a specific priority within the homecare services for years. Findings thus may not reflect other contexts, where prevention of falls might not be a topic of interest within the organization. However, we thoroughly described the context and participants, enabling readers to assess the transferability of the results. To ensure transparency in the study and research process, we adhered to the Standards for Reporting Qualitative Research (SRQR) (53) in our reporting (Supplementary File 2).

## 5 Conclusion

In this study we explored the experiences of managers’ and perceptions of their roles in implementation of fall prevention interventions in homecare services. Interpreting our results in light of existing literature, it becomes clear that there is a need to define and achieve an aligned understanding of managers’ roles in the implementation process. Managers described influencing implementation by using existing workflows and fostering a positive attitude among employees. They emphasized the value of trust, flexibility in the process, and listening and involving employees in the implementation. Recognizing that implementation can be challenging, managers found supportive systems for long-term assistance to be crucial in the process. Knowledge from this study may benefit how managers in clinical practice can be involved in implementation and provide knowledge for researchers investigating implementation in homecare services.

## Data Availability

The data that support the findings of this study are available from the corresponding author upon reasonable request.
