# Supplementary material for: Managers’ perspectives on their role in implementing fall prevention interventions: a qualitative interview study in Norwegian homecare services
Source: Front Health Serv. 2024 Sep 27;4:1456028. doi: 10.3389/frhs.2024.1456028 (PMC11467783; doi:10.3389/frhs.2024.1456028)
Supplement: Supplementary file 1 [file Datasheet1.docx]

**Interview guide**

Welcome from the interviewer: The aim of this interview is to explore your experiences and perceptions of your role in the implementation of fall prevention interventions. You have already red the information paper and signed an informed consent for participation. Just as a repetition, it is voluntary to participate, and you can withdraw you consent at any time. The session will be recorded, so please remember not to share sensitive information about persons not present.

Do you have any questions before we begin?

**Questions to managers**

- Can you tell me a bit about the department you are managing and your role as a manager?
- Fall prevention is related to identifying falls, conducting multifactorial assessments, and providing interventions such as medication reviews or adjustments in the home. Can you talk about how your department works with fall prevention?
- Can you talk about what your role is in the implementation of fall prevention?
- How would you describe the optimal scenario for implementing fall prevention?
- One challenge in practice is achieving sustainable changes. What does it take to make sustainable changes in your department?

**Questions to managers in the Co-creation study**

- Could you tell us about your role as a manager and how you focus on fall prevention?
- Managers' roles have previously been suggested as important for the implementation of fall prevention. What are your thoughts on this?
- Can you describe how you facilitate collaboration?
- How do you provide competence enhancement on fall prevention?
- How do you facilitate achieving sustainable changes?
- What are your thoughts on how to succeed in implementation?
- One challenge in implementation is motivation and engagement among employees. What are your experiences with this?

**Questions to managers from the Feasibility study**

- Can you describe the culture for change in your department?
- Can you describe how implementation activities are integrated into daily practice?
- How do you facilitate implementation?
- What do you consider to be the most challenging when implementing fall prevention?
- What is your role in implementation in general?
- What is your most important role in implementing fall prevention?

Finally, the interviewer will provide a summary of the key points and check with the participant to ensure understanding. To open the floor for additional information, the interview concludes by asking, "Is there anything I haven't asked about that you think is important, or that you would like to add?"
